# ﻿An updated catalogue of biting midges of the genus *Culicoides* Latreille, 1809 (Diptera, Ceratopogonidae) of Mexico and their known distribution by state

**DOI:** 10.3897/zookeys.1167.102858

**Published:** 2023-06-14

**Authors:** Alejandro Mendez-Andrade, Sergio Ibáñez-Bernal

**Affiliations:** 1 Instituto de Ecología, A.C. Red Ambiente y Sustentabilidad. Carretera antigua a Coatepec 351, Col. El Haya, Xalapa, Veracruz, C.P. 91073, Mexico Instituto de Ecología, A. C. Red Ambiente y Sustentabilidad Xalapa Mexico

**Keywords:** Biting midges, Culicoidini, distribution, hematophagy, species list

## Abstract

An updated catalogue of *Culicoides* of Mexico is presented. It includes 86 species with their regional distribution and corresponding record references, known immature stages and associated pathogens. In addition, a taxonomic key for subgenera and species groups for Mexico is presented and an index of species by state is included.

## ﻿Introduction

Species of the genus *Culicoides* Latreille, 1809 are tiny hematophagous dipterans, between 1 to 3 millimeters of length, and are known as “jejenes”, “polvorines”, “purrujas”, or “chaquistes” in Spanish, and as “biting midges”, “no-see-ums”, or “punkies” in English (Ibáñez-Bernal et al. 1996; Spinelli et al. 2005). This genus is the most diverse in the family Ceratopogonidae and is composed of 1,368 known extant species, classified into 33 subgenera, 38 species groups, and the remainder as miscellaneous (Borkent and Dominiak 2020; Borkent et al. 2022; Chatterjee et al. 2022; Labuschagne et al. 2023; Sarkar et al. 2023). Except for the regions of New Zealand and Antarctica, biting midges are distributed throughout the world and their populations occur in wetland, forest, agricultural, rural, and peri-urban areas, from sea level to 4,200 m of altitude (Wirth and Blanton 1959; Harrup et al. 2015).

Biting midges are a global concern because they cause direct and indirect damage to humans, domestic animals, and wildlife. Some species are vectors of pathogens, including viruses, bacteria, protozoa, and filariae (Vargas 1969; Borkent 2004) that infect different vertebrates, mainly birds and ruminant mammals, and produce important diseases such as Oropouche fever, Bluetongue disease, African horse sickness, Epizootic hemorrhagic disease, Schmallenberg disease (Mellor et al. 2000; Sick et al. 2019), as well as avian malaria by different species of *Haemoproteus* Kruse, 1890, *Leucocytozoon* Berestneff, 1904, *Hepatocystis* Levaditi & Schoen, 1932, and *Trypanosoma* Gruby, 1843 (Valkiunas 2005). In addition, their high densities and often-irritating bites cause skin lesions, secondary infections, and allergies (Blanton and Wirth 1979; Ibáñez-Bernal et al. 2020) and can generate important economic losses in sectors related to recreation and tourism, forestry, and agriculture (Wirth and Blanton 1974; Borkent and Spinelli 2007).

In several regions of the world, the transmission of pathogens by different species of *Culicoides* directly affects human health and has a detrimental effect on the farming industry and wildlife conservation programs. Mansonellosis, caused by *Mansonella* filariae, affects regions in America and Africa, and Oropouche fever, caused by a virus of the same name affects Central and South American countries, both considered neglected diseases which are the most relevant human diseases in which a *Culicoides* species is involved as vector (Linley et al. 1983; Borkent 2004; Mediannikov and Ranque 2018; Romero-Alvarez and Escobar 2018). However, pathogens of veterinary importance, such as Bluetongue virus, Epizootic hemorrhagic virus, Schmallenberg virus, and avian haemosporidians, cause significant economic losses at local and regional levels in farm industry (Mills et al. 2017; Alkhamis et al. 2020; Marzal and García-Longoria 2020), mainly because of high animal mortality and morbidity, transport bans, trade restrictions, prevention and control costs, and management and conservation efforts directed at wildlife (Sick et al. 2019; Marzal and García-Longoria 2020).

Despite the important sanitary and economic damages caused by different species of *Culicoides*, in Mexico, they have been scarcely studied as compared with other hematophagous Diptera; given their global relevance, at present, the study of the genus in the country can be considered neglected. The current knowledge of Mexican species corresponds to the taxonomic description (Root and Hoffman 1937; Hoffman 1939; Vargas 1944, 1953a, 1953b, 1954, 1955, 1960, 1972; Macfie 1948; Vargas and Wirth 1955; Spinelli and Huerta 2015) and distribution records (Wirth and Jones 1957; Wirth and Hubert 1960; Wirth and Blanton 1978; Wirth and Moraes 1979; Huerta 1996; Huerta et al. 2012, 2020, 2022), progressively compiled in different lists and catalogues.

The first list of species from Mexico was elaborated by Vargas (1945) and included 14 species without a subgeneric classification. A decade later, the known richness was increased to 31 species by Fox (1955) in his catalog of hematophagous ceratopogonids of America and, later, to 50 species by Wirth (1974) in the "Catalog of the Diptera of America south of the United States", both of which already present a subgeneric classification of the genus. Ibáñez-Bernal et al. (1996) updated the richness of the family Ceratopogonidae in Mexico and specifically for the genus *Culicoides*, reporting 71 species, 13 subgenera, and eight endemic species; however, the identity of the species was not mentioned. The most recent catalog that most completely incorporated the known *Culicoides* fauna of Mexico, and its regional distribution was presented by Borkent and Spinelli (2000), in which they reported 69 species (eight endemic), 14 subgenera, and eight species groups. More recently, catalogues of the family Ceratopogonidae have corresponded to the Nearctic (Borkent and Grogan 2009) and Neotropical fauna (Borkent and Spinelli 2007), the two biogeographic regions that collide in Mexico (Griffiths 1980; Halffter 2003; Morrone 2005), but do not include all species known from Mexico. Particularly for the Neotropical Region, 49 species, 12 subgenera and 7 species groups have been reported, and for the Nearctic Region, 38 species, 10 subgenera and 3 species groups, respectively; neither of the two catalogues included the known endemic species of the country which were unknown at the time.

Mexico’s biogeographic regions are important for *Culicoides* species distribution. The Nearctic and Neotropical Regions possess different habitats, with arid xerophyte vegetation and temperate forests in the north, and tropical forests in the south (Griffiths 1980). *Culicoides* species can be restricted to one region or occur in both. The “Mexican Transition Zone” is a vital area with various ecosystems and serving as a bridge between the two regions (Halffter 2003). It allows for the exchange of flora and fauna, and both regions share some biotic elements due to historical and ecological processes, but also there are conditions that promote endemism.

In recent years, the emergence and re-emergence of diseases caused by pathogens transmitted by different *Culicoides* species, such as the Schmallenberg virus and Bluetongue virus (Sick et al. 2019), has increased the interest of researchers and institutions and the need for their study worldwide. Vector species of *Culicoides* have already been reported in Mexico (Dampf 1936; Borkent and Spinelli 2007); however, their role as vectors in the country is uncertain. Mexico hosts an important vertebrate faunal diversity, the four North American migratory bird routes, and the largest migration of birds of prey in the world (Rappole et al. 1998) making it an important region where pathogens can be dispersed and maintained by biting midges, increasing the risk of emergence or re-emergence of zoonoses or wildlife diseases.

Vector-borne diseases are increasing their distribution and incidence, acquiring a more preponderant role in the maintenance of human welfare. Under this framework and with the scarce attention that historically has been given to the study of *Culicoides* in Mexico, it is evident and necessary to update the known information of the genus in the country. This work presents an update catalogue of the *Culicoides* of Mexico. It includes 86 species of 15 subgenera, ten species groups and four species not included in any group or subgenus (Table 1). It is arranged by subgenus and species, followed by the author(s) and the year of publication of the original description, their synonymies, and references. Subgenera and species groups are presented alphabetically, but species are classified according to the most recent proposal of Borkent and Dominiak (2020) and the assignment to the *limai* group of *C.luglani* by Phillips (2022). For each species the original reference, type locality, and their regional distribution by state, with the corresponding record reference are also presented. In addition, a key for subgenera and species groups and an index of species by state are presented.

**Table 1.** Number of species of Culicoides by subgenus and species group in Mexico.

Subgenus/species group	Number of species
Subgenus Amossovia	3
Subgenus Anilomyia	3
Subgenus Avaritia	3
Subgenus Beltranmyia	1
Subgenus Culicoides	5
Subgenus Diphaomyia	4
Subgenus Drymodesmyia	14
Subgenus Glaphiromyia	3
Subgenus Haematomyidium	5
Subgenus Hoffmania	9
Subgenus Macfiella	2
Subgenus Mataemyia	1
Subgenus Monoculicoides	3
Subgenus Oecacta	3
Subgenus Selfia	2
Subgenus unplaced, *acotylus* species group	1
Subgenus unplaced, *daedalus* species group	3
Subgenus unplaced, *eublepharus* species group	3
Subgenus unplaced, *fluvialis* species group	2
Subgenus unplaced, *leoni* species group	3
Subgenus unplaced, *limai* species group	1
Subgenus unplaced, *mohave* species group	4
Subgenus unplaced, *reticulatus* species group	1
Subgenus unplaced, *stigmalis* species group	1
Subgenus unplaced, *stonei* species group	2
Species of Culicoides unplaced to subgenus or species group	4
Total	86

## ﻿Genus *Culicoides* Latreille, 1809

**Subgenus Amossovia Glukhova, 1989**

*Amossovia* Glukhova, 1989: 226 (as subgenus of *Culicoides*). Type species: *Culicoidesdendrophilus* Amosova, by original designation.

**Culicoides (Amossovia) cochisensis Wirth & Blanton, 1967**

*Culicoidescochisensis* Wirth & Blanton, 1967: 216. Type locality: United States, Arizona, Santa Cruz, Sycamore Canyon. Additional references: Wirth (1974), Wirth et al. (1985, 1988), Borkent and Wirth (1997), Borkent and Spinelli (2000), Borkent and Grogan (2009).

**Distribution in Mexico.** Baja California Sur (Wirth and Blanton 1967).

**General distribution.** Nearctic. USA, Mexico (Borkent and Grogan 2009).

**Immature stages.** Larva (Murphree and Mullen 1991).

**Associated pathogens.** Unknown.

**Culicoides (Amossovia) oklahomensis Khalaf, 1952**

*Culicoidesoklahomensis* Khalaf, 1952: 355 (as subspecies of *villosipennis* Root and Hoffman). Type locality: United States, Oklahoma, Wichita Refuge. Additional references: Wirth (1965, 1974), Wirth et al. (1985, 1988), Borkent and Wirth (1997), Borkent and Spinelli (2000), Borkent and Grogan (2009).

**Distribution in Mexico.** Baja California, Sonora (Wirth and Blanton 1967).

**General distribution.** Nearctic. USA, Mexico. Neotropical. Guatemala (Borkent and Grogan 2009).

**Immature stages.** Larva (Murphree and Mullen 1991), pupa (Lamberson et al. 1992).

**Associated pathogens.** Unknown.

**Culicoides (Amossovia) ousairani Khalaf, 1952**

*Culicoidesousairani* Khalaf, 1952: 354. Type locality: United States, Oklahoma, Wichita Refuge. Additional references: Wirth (1965, 1974), Blanton and Wirth (1979), Wirth et al. (1985, 1988), Borkent and Wirth (1997), Borkent and Spinelli (2000), Borkent and Grogan (2009).

**Distribution in Mexico.** Nuevo León (Wirth and Blanton 1967).

**General distribution.** Nearctic. USA, Mexico (Borkent and Grogan 2009).

**Immature stages.** Larva (Murphree and Mullen 1991, pupa (Lamberson et al. 1992).

**Associated pathogens.** Unknown.

### ﻿Subgenus Anilomyia Vargas, 1960

*Anilomyia* Vargas, 1960: 37 (as subgenus of *Culicoides*). Type species: *Culicoidescovagarciai* Ortiz, by original designation.

**Culicoides (Anilomyia) hayesi Matta, 1967**

*Culicoideshayesi* Matta, 1967: 75. Type locality: Honduras, Distrito Central, La Tigra. Additional references: Wirth (1974), Wirth et al. (1988), Borkent and Wirth (1997), Borkent and Spinelli (2000, 2007).

**Distribution in Mexico.** Tamaulipas (Wirth and Blanton 1970).

**General distribution.** Neotropical. Mexico, Honduras (Borkent and Spinelli 2007).

**Immature stages.** Larva, pupa (Matta 1967).

**Associated pathogens.** Unknown.

**Culicoides (Anilomyia) nigrigenus Wirth & Blanton, 1956**

*Culicoidesnigrigenus* Wirth & Blanton, 1956b: 222. Type locality: Panama, Boca del Toro, Almirante. Additional references: Wirth (1974), Aitken et al. (1975), Wirth et al. (1988), Borkent and Wirth (1997), Borkent and Spinelli (2000, 2007).

**Distribution in Mexico.** Veracruz (Wirth and Blanton 1970), Hidalgo (Huerta et al. 2012).

**General distribution.** Neotropical. Mexico, Guatemala, Belize, El Salvador, Nicaragua, Panama, Colombia, Trinidad and Tobago, Argentina (Borkent and Spinelli 2007).

**Immature stages.** Unknown.

**Associated pathogens.** Unknown.

**Culicoides (Anilomyia) pseudodecor Spinelli & Huerta, 2015**

*Culicoidespseudodecor* Spinelli & Huerta, 2015: 818. Type locality: Mexico, Morelos, El Salto Falls.

**Distribution in Mexico.** Morelos, Veracruz (Spinelli and Huerta 2015).

**General distribution.** Endemic. Mexico. (Spinelli and Huerta 2015).

**Immature stages.** Unknown.

**Associated pathogens.** Unknown.

### ﻿Subgenus Avaritia Fox, 1955

*Avaritia* Fox, 1955: 218 (as subgenus of *Culicoides*). Type species: *Ceratopogonobsoletus* Meigen, by original designation.

**Culicoides (Avaritia) boydi Wirth & Mullens, 1992**

*Culicoidesboydi* Wirth & Mullens, 1992: 1006. Type locality: United States, California Riverside County, Deep Canyon, Santa Rosa Mountains. Additional references: Borkent and Spinelli (2000), Borkent and Grogan (2009).

**Distribution in Mexico.** Baja California (Wirth and Mullens 1992).

**General distribution.** Nearctic. USA, Mexico (Borkent and Grogan 2009).

**Immature stages.** Egg, larva, pupa (Breidenbaugh and Mullens 1999b).

**Associated pathogens.** Bluetongue virus (Wirth and Mullens 1992).

**Culicoides (Avaritia) pusilloides Wirth & Blanton, 1955**

*Culicoidespusilloides* Wirth & Blanton, 1955a: 104. Type locality: Panama, Boca del Toro Province, Almirante. Additional references: Wirth and Blanton (1959, 1974), Wirth (1974), Aitken et al. (1975), Wirth et al. (1988), Borkent and Wirth (1997), Borkent and Spinelli (2000, 2007).

**Distribution in Mexico.** Chiapas (Huerta et al. 2012).

**General distribution.** Neotropical. Mexico, Guatemala, Belize, Panama (Borkent and Spinelli 2007).

**Immature stages.** Unknown.

**Associated pathogens.** Unknown.

**Culicoides (Avaritia) pusillus Lutz, 1913**

*Culicoidespusillus* Lutz, 1913: 52. Type locality: Brazil, Rio de Janeiro, Manguinhos. Additional references: Fox (1955), Forattini (1957), Wirth and Blanton (1959, 1974), Wirth (1965, 1974), Aitken et al. (1975), Blanton and Wirth (1979), Wirth et al. (1985), Borkent and Wirth (1997), Borkent and Spinelli (2000, 2007), Borkent and Grogan (2009).

**Distribution in Mexico.** Chiapas (Macfie 1948), Tabasco, Veracruz (Huerta et al. 2012), Oaxaca (Huerta et al. 2020).

**General distribution.** Nearctic. USA. Neotropical. Mexico, Central and South America (Borkent and Spinelli 2007).

**Immature stages.** Unknown.

**Associated pathogens.** Bluetongue virus (Mo et al. 1994).

### ﻿Subgenus Beltranmyia Vargas, 1953

*Beltranmyia* Vargas, 1953: 34 (as subgenus of *Culicoides*). Type species: *Culicoidescrepuscularis* Malloch, by original designation.

**Culicoides (Beltranmyia) crepuscularis Malloch, 1915**

*Culicoidescrepuscularis* Malloch, 1915: 303. Type locality: United States, Illinois, Du Bois. Additional references: Fox (1955), Wirth (1965, 1974), Blanton and Wirth (1979), Wirth et al. (1985, 1988), Borkent and Wirth (1997), Borkent and Spinelli (2000, 2007), Borkent and Grogan (2009).

**Distribution in Mexico.** Mexico City (Root and Hoffman 1937; Vargas 1945; formerly Distrito Federal), Morelos, Sonora, Veracruz (Wirth et al. 1988), Coahuila (Huerta et al. 2012).

**General distribution.** Nearctic. Canada, USA, Mexico. Neotropical. Mexico, El Salvador, Honduras, Costa Rica (Borkent and Spinelli 2007).

**Immature stages.** Larva, pupa (Jamnback 1965).

**Associated pathogens.***Chandlerellaquiscali* (Robinson 1971), *Eufilarialongicaudata* (Hibler 1963), *Haemoproteusdanilewskyi* (Fallis and Bennett 1960), *H.fringillae* (Fallis and Bennet 1961), *H.velans* (Borkent 2004).

### ﻿Subgenus Culicoides Latreille, 1809

*Silvicola* Mirzaeva & Isaev, 1990: 98 (as subgenus of *Culicoides*). Type species: *Culicoidesgrisescens* Edwards, by original designation.

**Culicoides (Culicoides) elutus Macfie, 1948**

*Culicoideselutus* Macfie, 1948: 75. Type locality: Mexico, Chiapas, El Carrizal. Additional references: Fox (1955), Forattini (1957), Wirth and Blanton (1959), Wirth (1974), Wirth et al. (1988), Borkent and Wirth (1997), Borkent and Spinelli (2000, 2007).

**Distribution in Mexico.** Oaxaca (Vargas 1945, as Culicoideselutusvar.cockerelliCoquillett 1901), Chiapas (Macfie 1948).

**General distribution.** Neotropical. Mexico, Belize, Guatemala, Honduras, El Salvador, Nicaragua, Costa Rica, Panama (Borkent and Spinelli 2007).

**Immature stages.** Unknown.

**Associated pathogens.** Unknown.

**Culicoides (Culicoides) fortinensis Spinelli & Huerta, 2015**

*Culicoidesfortinensis* Spinelli & Huerta, 2015: 812. Type locality: Mexico, Veracruz, Fortín de la Flores.

**Distribution in Mexico.** Veracruz (Spinelli and Huerta 2015).

**General distribution.** Endemic. Mexico (Spinelli and Huerta 2015).

**Immature stages.** Unknown.

**Associated pathogens.** Unknown.

**Culicoides (Culicoides) luteovenus Root & Hoffman, 1937**

*Culicoidesluteovenus* Root & Hoffman, 1937: 156. Type locality: Mexico, Mexico City, San Jacinto. Additional references: Fox (1955), Forattini (1957), Wirth and Blanton (1959), Wirth (1965, 1974), Wirth et al. (1988), Borkent and Wirth (1997), Borkent and Spinelli (2000, 2007).

**Distribution in Mexico.** Mexico City (Root and Hoffman 1937), Oaxaca (Vargas 1945), Chiapas (Macfie 1948), Veracruz (Huerta et al. 2012).

**General distribution.** Nearctic. USA. Neotropical. Mexico, Belize, Guatemala, El Salvador, Honduras, Nicaragua, Costa Rica, Panama (Borkent and Spinelli 2007).

**Immature stages.** Larva, pupa (Wirth 1952).

**Associated pathogens.** Unknown.

**Culicoides (Culicoides) neopulicaris Wirth, 1955**

*Culicoidesneopulicaris* Wirth, 1955: 355. Type locality: United States, Texas, Kerrville. Additional references: Wirth (1965, 1974), Wirth et al. (1985, 1988), Borkent and Wirth (1997), Borkent and Spinelli (2000, 2007), Borkent and Grogan (2009), Huerta et al. (2012).

**Distribution in Mexico.** San Luis Potosí, Guerrero (Wirth 1955), Chiapas, Morelos, Veracruz (Wirth and Blanton 1969), Hidalgo, Estado de México, Yucatán (Huerta et al. 2012), Oaxaca (Huerta et al. 2020).

**General distribution.** Nearctic. USA. Neotropical. Mexico, Belize, Guatemala, Honduras, El Salvador, Nicaragua, Costa Rica (Borkent and Spinelli 2007).

**Immature stages.** Pupa (Jones 1961).

**Associated pathogens.** Unknown.

**Culicoides (Culicoides) rulfoi Spinelli & Huerta, 2015**

*Culicoidesrulfoi* Spinelli & Huerta, 2015: 816. Type locality: Mexico, Michoacán, Puerto Garnica.

**Distribution in Mexico**: Michoacán (Spinelli and Huerta 2015).

**General distribution.** Endemic. Mexico (Spinelli and Huerta 2015).

**Immature stages.** Unknown.

**Associated pathogens.** Unknown.

### ﻿Subgenus Diphaomyia Vargas, 1960

*Diphaomyia* Vargas, 1960: 40 (as subgenus of *Culicoides*). Type species: *Culicoidesbaueri* Hoffman, by original designation.

**Culicoides (Diphaomyia) baueri Hoffman, 1925**

*Culicoidesbaueri* Hoffman, 1925: 297. Type locality: United States, Maryland, Baltimore. Additional references: Root and Hoffman (1937), Vargas (1945), Fox (1955), Vargas and Wirth (1955), Forattini (1957), Wirth (1965, 1974), Wirth et al. (1985), Borkent and Wirth (1997), Borkent and Grogan (2009).

**Distribution in Mexico.** Puebla (Huerta et al. 2012), Oaxaca (Huerta et al. 2020).

**General distribution.** Nearctic. USA. Neotropical. Mexico (Borkent and Grogan 2009).

**Immature stages.** Larva (Murphree and Mullen 1991).

**Associated pathogens.***Haemoproteusmansoni* (as *H.meleagridis*, Atkinson et al. 1983).

**Culicoides (Diphaomyia) blantoni Vargas & Wirth, 1955**

*Culicoidesblantoni* Vargas & Wirth, 1955: 33. Type locality: Mexico, Tamaulipas, Ciudad Mante. Additional references: Wirth (1965, 1974), Wirth et al. (1985, 1988), Borkent and Wirth (1997), Borkent and Spinelli (2000, 2007), Borkent and Grogan (2009).

**Distribution in Mexico.** Guerrero, Puebla, San Luis Potosí, Tamaulipas (Vargas and Wirth 1955), Morelos, Sinaloa (Wirth 1974), Veracruz (Huerta et al. 2012), Tabasco (Huerta et al. 2022).

**General distribution.** Nearctic. USA. Neotropical. Mexico (Borkent and Grogan 2009).

**Immature stages.** Unknown.

**Associated pathogens.** Unknown.

**Culicoides (Diphaomyia) haematopotus Malloch, 1915**

*Culicoideshaematopotus* Malloch, 1915: 302. Type locality: United States, Illinois. Additional references: Vargas (1949), Fox (1955), Wirth (1965, 1974), Atchley and Wirth (1979), Blanton and Wirth (1979), Wirth et al. (1985, 1988), Borkent and Wirth (1997), Borkent and Spinelli (2000, 2007), Borkent and Grogan (2009).

**Distribution in Mexico.** Mexico City (Root and Hoffman 1937), Guerrero (Vargas 1945), Chiapas (Macfie 1948), Baja California (Atchley and Wirth 1979), Puebla, Veracruz (Huerta et al. 2012).

**General distribution.** Nearctic. USA, Mexico. Neotropical. Mexico, Belize, Guatemala, Honduras (Borkent and Grogan 2009).

**Immature stages.** Larva (Jamnback 1965), pupa (Thomsen, 1937).

**Associated pathogens.***Chandlerellaquiscali* (Robinson 1971), *Chandlerellastriatospicula*, *Eufilarialongicaudata* (Hibler 1963), *Haemaproteusmansoni* (as *H.meleagridis*, Atkinson et al. 1988), Bluetongue virus (Becker et al. 2010).

**Culicoides (Diphaomyia) iriartei Fox, 1952**

*Culicoidesiriartei* Fox, 1952: 368. Type locality: Venezuela, Zulia, La Salina. Additional references: Wirth and Blanton (1959), Wirth (1974), Wirth et al. (1988), Borkent and Wirth (1997), Borkent and Spinelli (2000, 2007).

*Culicoidesvargasi* Wirth & Blanton, 1953: 74, syn. Type locality: Panama.

**Distribution in Mexico.** Chiapas, Veracruz (Huerta et al. 2012).

**General distribution.** Neotropical. Mexico, Guatemala, Belize, El Salvador, Nicaragua, Costa Rica, Panama, Colombia, Venezuela, Trinidad and Tobago, Brazil (Borkent and Spinelli 2007).

**Immature stages.** Unknown.

**Associated pathogens.** Unknown.

### ﻿Subgenus Drymodesmyia Vargas, 1960

*Drymodesmyia* Vargas, 1960: 40 (as subgenus of *Culicoides*). Type species: *Culicoidescopiosus* Root and Hoffman, by original designation.

**Culicoides (Drymodesmyia) arizonensis Wirth & Hubert, 1960**

*Culicoidesarizonensis* Wirth & Hubert, 1960: 655. Type locality: United States, Arizona, Maricopa. Additional references: Wirth (1965, 1974), Wirth et al. (1985, 1988), Borkent and Wirth (1997), Borkent and Spinelli (2000), Borkent and Grogan (2009).

**Distribution in Mexico.** Baja California (Wirth and Hubert 1960).

**General distribution.** Nearctic. USA, Mexico (Borkent and Grogan 2009).

**Immature stages.** Unknown.

**Associated pathogens.** Unknown.

**Culicoides (Drymodesmyia) bakeri Vargas, 1954**

*Culicoidesbakeri* Vargas, 1954: 27. Type locality: Mexico, Mexico City (formerly Distrito Federal), Chapultepec. Additional references: Wirth et al. (1988), Borkent and Wirth (1997), Borkent and Spinelli (2000).

**Distribution in Mexico.** Mexico City (Vargas 1954).

**General distribution.** Endemic. Mexico (Borkent and Spinelli 2000).

**Immature stages.** Unknown.

**Associated pathogens.** Unknown.

**Culicoides (Drymodesmyia) butleri Wirth & Hubert, 1960**

*Culicoidesbutleri* Wirth & Hubert, 1960: 650. Type locality: United States, Arizona, Baboquivari, Brown Canyon. Additional references: Wirth et al. (1985, 1988), Borkent and Spinelli (2000), Borkent and Grogan (2009).

**Distribution in Mexico.** Nuevo León (Wirth et al. 1988).

**General distribution.** Nearctic. USA, Mexico (Borkent and Grogan 2009).

**Immature stages.** Unknown.

**Associated pathogens.** Unknown.

**Culicoides (Drymodesmyia) cacticola Wirth & Hubert, 1960**

*Culicoidescacticola* Wirth & Hubert, 1960: 653. Type locality: United States, California, Los Angeles, San Dimas Canyon. Additional references: Wirth et al. (1985, 1988), Borkent and Wirth (1997), Borkent and Spinelli (2000), Borkent and Grogan (2009).

**Distribution in Mexico.** Baja California Sur, Sonora (Monarch 2022).

**General distribution.** Nearctic. USA, Mexico (Borkent and Grogan 2009).

**Immature stages.** Larva (Murphree and Mullen 1991), egg, pupa (Breidenbaugh and Mullens 1999b).

**Associated pathogens.** Unknown.

**Culicoides (Drymodesmyia) copiosus Root & Hoffman, 1937**

*Culicoidescopiosus* Root & Hoffman, 1937: 171. Type locality: Mexico, Mexico City (formerly Distrito Federal), San Jacinto. Additional references: Vargas (1945), Fox (1955), Wirth (1965, 1974), Wirth et al. (1985, 1988), Borkent and Wirth (1997), Borkent and Spinelli (2000), Borkent and Grogan (2009).

**Distribution in Mexico.** Mexico City (Root and Hoffman 1937), Baja California (Wirth and Hubert 1960).

**General distribution.** Nearctic. USA, Mexico (Borkent and Grogan 2009).

**Immature stages.** Unknown.

**Associated pathogens.** Unknown.

**Culicoides (Drymodesmyia) insolatus Wirth & Hubert, 1960**

*Culicoidesinsolatus* Wirth & Hubert, 1960: 654. Type locality: Mexico, Baja California, San Felipe. Additional references: Wirth (1974), Wirth et al. (1985, 1988), Borkent and Wirth (1997), Borkent and Spinelli (2000), Borkent and Grogan (2009).

**Distribution in Mexico.** Baja California (Wirth and Hubert 1960).

**General distribution.** Nearctic. USA, Mexico (Borkent and Grogan 2009).

**Immature stages.** Unknown.

**Associated pathogens.** Unknown.

**Culicoides (Drymodesmyia) jamaicensis Edwards, 1922**

*Culicoidesjamaicensis* Edwards, 1922: 165 (as var.loughnani Edwards). Type locality: Jamaica, Kingston. Additional references: Fox (1955), Wirth and Blanton (1959, 1974), Wirth and Hubert (1960), Wirth (1965, 1974), Aitken et al. (1975), Wirth et al. (1985, 1988), Borkent and Wirth (1997), Borkent and Spinelli (2000, 2007), Borkent and Grogan (2009), Huerta et al. (2020).

**Distribution in Mexico.** Chiapas (as var.loughnaniMacfie 1948), Veracruz (Wirth and Hubert 1960), Yucatán (Borkent and Spinelli 2007), Guerrero, Estado de México, Jalisco, Oaxaca (Huerta et al. 2012), Tabasco (Huerta et al. 2022).

**General distribution.** Nearctic. USA, Mexico. Neotropical. Mexico, Belize, Guatemala, El Salvador, Honduras, Nicaragua, Costa Rica, Panama, Colombia, Venezuela (Borkent and Spinelli 2007).

**Immature stages.** Unknown.

**Associated pathogens.** Unknown.

**Culicoides (Drymodesmyia) loughnani Edwards, 1922**

*Culicoidesloughnani* Edwards, 1922: 165. Type locality: Jamaica, Kingston. Additional references: Wirth (1965, 1974), Wirth et al. (1985, 1988), Borkent and Wirth (1997), Borkent and Spinelli (2000, 2007), Borkent and Grogan (2009).

**Distribution in Mexico.** Yucatán (Borkent and Spinelli 2007).

**General distribution.** Nearctic. USA. Neotropical. Mexico, Bahamas, Cuba, Jamaica. Australian. Australia (Borkent and Grogan 2009).

**Immature stages.** Unknown.

**Associated pathogens.** Unknown.

**Culicoides (Drymodesmyia) panamensis Barbosa, 1947**

*Culicoidespanamensis* Barbosa, 1947: 22. Type locality: Panama, Barro Colorado. Additional references: Fox (1955), Wirth (1955, 1974), Forattini (1957), Wirth and Blanton (1959, 1974), Wirth et al. (1988), Borkent and Wirth (1997), Borkent and Spinelli (2000, 2007).

*Culicoidesalambiculorum* Macfie, 1948: 81, syn. Type locality: Mexico, Chiapas.

**Distribution in Mexico.** Chiapas (Macfie 1948), Nayarit, Veracruz (Borkent and Spinelli 2000), Baja California, Estado de México, Morelos (Huerta et al. 2012).

**General distribution.** Neotropical. Mexico, Belize, Guatemala, El Salvador, Honduras, Costa Rica, Jamaica (Borkent and Spinelli 2007).

**Immature stages.** Unknown.

**Associated pathogens.** Unknown.

**Culicoides (Drymodesmyia) poikilonotus Macfie, 1948**

*Culicoidespoikilonotus* Macfie, 1948: 82. Type locality: Mexico, Chiapas, El Vergel. Additional references: Fox (1955), Forattini (1957), Wirth and Blanton (1959), Wirth (1974), Aitken et al. (1975), Wirth et al. (1988), Borkent and Wirth (1997), Borkent and Spinelli (2000, 2007).

*Culicoidescacozelus* Macfie, 1948: 85, syn. Type locality: Mexico, Chiapas.

*Culicoideshertigi* Wirth & Blanton, 1953: 229, syn. Type locality: Panama.

**Distribution in Mexico.** Chiapas (Macfie 1948), Veracruz (Huerta et al. 2012), Tabasco (Huerta et al. 2022).

**General distribution.** Neotropical. Mexico, Belize, Guatemala, El Salvador, Honduras, Nicaragua, Costa Rica, Panama, Venezuela, Trinidad and Tobago, Brazil (Borkent and Spinelli 2007).

**Immature stages.** Unknown.

**Associated pathogens.** Unknown.

**Culicoides (Drymodesmyia) ryckmani Wirth & Hubert, 1960**

*Culicoidesryckmani* Wirth & Hubert, 1960: 656. Type locality: United States, California, Los Angeles, San Dimas Canyon. Additional references: Wirth (1965, 1974), Wirth et al. (1988), Borkent and Wirth (1997), Borkent and Spinelli (2000), Borkent and Grogan (2009).

**Distribution in Mexico.** Baja California (Wirth and Hubert 1960).

**General distribution.** Nearctic. USA, Mexico (Borkent and Grogan 2009).

**Immature stages.** Unknown.

**Associated pathogens.** Unknown.

**Culicoides (Drymodesmyia) sitiens Wirth & Hubert, 1960**

*Culicoidessitiens* Wirth & Hubert, 1960: 652. Type locality: United States, California, Los Angeles, San Dimas Canyon. Additional references: Atchley (1967), Wirth (1965, 1974), Wirth et al. (1985), Borkent and Wirth (1997), Borkent and Spinelli (2000), Borkent and Grogan (2009).

**Distribution in Mexico.** Baja California (Wirth and Hubert 1960).

**General distribution.** Nearctic. USA, Mexico (Borkent and Grogan 2009).

**Immature stages.** Unknown.

**Associated pathogens.** Unknown.

**Culicoides (Drymodesmyia) torridus Wirth & Hubert, 1960**

*Culicoidestorridus* Wirth & Hubert, 1960: 654. Type locality: Mexico, Baja California, San Felipe. Additional references: Wirth (1974), Wirth et al. (1985, 1988), Borkent and Wirth (1997), Borkent and Spinelli (2000), Borkent and Grogan (2009).

**Distribution in Mexico.** Baja California (Wirth and Hubert 1960).

**General distribution.** Nearctic. USA, Mexico (Borkent and Grogan 2009).

**Immature stages.** Unknown.

**Associated pathogens.** Unknown.

**Culicoides (Drymodesmyia) wirthomyia Vargas, 1953**

*Culicoideswirthomyia* Vargas, 1953: 227. Type locality: Mexico, Guerrero, Iguala. Additional references: Wirth (1974), Wirth et al. (1988), Borkent and Wirth (1997), Borkent and Spinelli (2000, 2007).

**Distribution in Mexico.** Guerrero (Vargas 1953b).

**General distribution.** Endemic. Mexico (Borkent and Spinelli 2000).

**Immature stages.** Unknown.

**Associated pathogens.** Unknown.

### ﻿Subgenus Glaphiromyia Vargas, 1960

*Glaphiromyia* Vargas, 1960: 41 (as subgenus of *Culicoides*). Type species: *Culicoidesscopus* Root and Hoffman, by original designation.

**Culicoides (Glaphiromyia) dampfi Root & Hoffman, 1937**

*Culicoidesdampfi* Root & Hoffman, 1937: 169. Type locality: Mexico, Mexico City (formerly Distrito Federal), San Jacinto. Additional references: Fox (1955), Wirth (1974), Wirth et al. (1988), Borkent and Wirth (1997), Borkent and Spinelli (2000).

**Distribution in Mexico.** Mexico City (Root and Hoffman 1937).

**General distribution.** Endemic. Mexico (Borkent and Spinelli 2000).

**Immature stages.** Unknown.

**Associated pathogens.** Unknown.

**Culicoides (Glaphiromyia) parascopus Wirth & Blanton, 1978**

*Culicoidesparascopus* Wirth & Blanton, 1978: 238. Type locality: Mexico, Michoacán, Puerto Garnica. Additional references: Wirth et al. (1988), Borkent and Wirth (1997), Borkent and Spinelli (2000, 2007).

**Distribution in Mexico.** Michoacán (Wirth and Blanton 1978).

**General distribution.** Endemic. Mexico (Borkent and Spinelli 2000).

**Immature stages.** Unknown.

**Associated pathogens.** Unknown.

**Culicoides (Glaphiromyia) scopus Root & Hoffman, 1937**

*Culicoidesscopus* Root & Hoffman, 1937: 170. Type locality: Mexico, Mexico City (formerly Distrito Federal), San Jacinto. Additional references: Vargas (1945), Fox (1955), Wirth and Blanton (1959), Wirth (1974), Wirth et al. (1988), Borkent and Wirth (1997), Borkent and Spinelli (2000, 2007).

**Distribution in Mexico.** Mexico City (Root and Hoffman 1937).

**General distribution.** Neotropical. Mexico, Costa Rica, Panama (Borkent and Spinelli 2007).

**Immature stages.** Unknown.

**Associated pathogens.** Unknown.

### ﻿Subgenus Haematomyidium Goeldi, 1905

*Haematomyidium* Goeldi, 1905: 137. Type species: *Haematomyidiumparaensis* Goeldi, by original designation.

**Culicoides (Haematomyidium) debilipalpis Lutz, 1913**

*Culicoidesdebilipalpis* Lutz, 1913: 60. Type locality: Brazil, São Paulo, Serra da Bocaina. Additional references: Macfie (1948), Fox (1955), Forattini (1957), Wirth (1965, 1974), Wirth et al. (1985), Borkent and Spinelli (2000, 2007), Borkent and Grogan (2009).

*Culicoideskhalafi* Beck, 1957: 104, syn. Type locality: United States, Florida.

*Culicoidesichesi* Ronderos & Spinelli, 1995: 77, syn. Type locality: Argentina, Misiones.

**Distribution in Mexico.** Veracruz, Yucatán (Huerta et al. 2012), Oaxaca (Huerta et al. 2020).

**General distribution.** Nearctic. USA. Neotropical. Mexico, Central and South America (Borkent and Grogan 2009).

**Immature stages.** Larva (Ronderos et al. 2010), pupa (Forattini 1957).

**Associated pathogens.** Bluetongue virus (Mullen et al. 1985).

**Culicoides (Haematomyidium) eadsi Wirth & Blanton, 1971**

*Culicoideseadsi* Wirth & Blanton, 1971a: 37. Type locality: United States, Texas, Cameron County. Additional references: Wirth (1974), Wirth et al. (1985, 1988), Borkent and Wirth (1997), Borkent and Spinelli (2000, 2007), Borkent and Grogan (2009).

**Distribution in Mexico.** Nayarit, San Luis Potosí, Sonora, Yucatán (Wirth and Blanton 1971a).

**General distribution.** Nearctic. USA. Neotropical. Mexico, Cuba, Guatemala (Borkent and Grogan 2009).

**Immature stages.** Unknown.

**Associated pathogens.** Unknown.

**Culicoides (Haematomyidium) ginesi Ortiz, 1951**

*Culicoidesginesi* Ortiz, 1951: 586. Type locality. Venezuela, San Felipe, Yaracuy. Additional references: Wirth and Blanton (1959), Wirth (1974), Wirth et al. (1988), Borkent and Spinelli (2000, 2007).

**Distribution in Mexico.** Oaxaca (Huerta et al. 2020).

**General distribution.** Neotropical. Mexico, El Salvador, Honduras, Nicaragua Costa Rica, Panama, Colombia, Venezuela, Trinidad and Tobago, Brazil, Argentina (Borkent and Spinelli 2007).

**Immature stages.** Unknown.

**Associated pathogens.** Unknown.

**Culicoides (Haematomyidium) kettlei Breidenbaugh & Mullens, 1999**

*Culicoideskettlei* Breidenbaugh & Mullens, 1999a: 150. Type locality: United States, California, Riverside County. Additional references: Borkent and Spinelli (2000), Borkent and Grogan (2009).

**Distribution in Mexico.** Baja California (Breidenbaugh and Mullens 1999a).

**General distribution.** Nearctic. USA, Mexico (Borkent and Grogan 2009).

**Immature stages.** Egg, larva, pupa (Breidenbaugh and Mullens 1999a).

**Associated pathogens.** Unknown.

**Culicoides (Haematomyidium) paraensis (Goeldi, 1905)**

*Culicoidesparaensis* (Goeldi, 1905): 137 (as *Haematomyidiumparaensis*). Type locality: Brazil, Pará. Additional references: Wirth (1965, 1974), Wirth and Blanton (1974), Blanton and Wirth (1979), Wirth et al. (1988), Borkent and Wirth (1997), Borkent and Spinelli (2000, 2007), Borkent and Grogan (2009), Huerta et al. (2022).

*Culicoidesundecimpunctatus* Kieffer, 1917: 307, syn. Type locality: Argentina, San Pablo.

**Distribution in Mexico.** Quintana Roo, San Luis Potosí (Blanton and Wirth 1979), Tabasco, Veracruz (Wirth and Felippe-Bauer 1989), Chiapas (Huerta et al. 2012).

**General distribution.** Nearctic. USA. Neotropical. Mexico, Central and South America (Borkent and Spinelli 2007).

**Immature stages.** Larva (Murphree and Mullen 1991), pupa (Lamberson et al. 1992).

**Associated pathogens.** Oropouche virus (Pinheiro et al. 1981).

### ﻿Subgenus Hoffmania Fox, 1948

*Hoffmania* Fox, 1948: 21 (as subgenus of *Culicoides*). Type species: *Culicoidesinamollae* Fox and Hoffman (= *Culicoidesinsignis* Lutz), by original designation.

**Culicoides (Hoffmania) diabolicus Hoffman, 1925**

*Culicoidesdiabolicus* Hoffman, 1925: 294. Type locality: Panama, Cabima. Additional references: Vargas (1945), Fox (1955), Wirth and Blanton (1959), Wirth (1974), Wirth et al. (1988), Borkent and Wirth (1997), Borkent and Spinelli (2000, 2007).

**Distribution in Mexico.** Chiapas, Veracruz (Vargas 1944), Oaxaca (Huerta et al. 2012).

**General distribution.** Neotropical. Mexico, Belize, Guatemala, El Salvador, Honduras, Nicaragua, Costa Rica, Panama, Venezuela, Ecuador (Borkent and Spinelli 2007).

**Immature stages.** Unknown.

**Associated pathogens.***Filaria* sp. (Dampf 1936).

**Culicoides (Hoffmania) filariferus Hoffman, 1939**

*Culicoidesfilariferus* Hoffman, 1939: 172. Type locality: Mexico, Chiapas, El Vergel. Additional references: Wirth (1974), Aitken et al. (1975), Wirth et al. (1988), Borkent and Wirth (1997), Borkent and Spinelli (2000, 2007).

**Distribution in Mexico.** Chiapas (Hoffman 1939), Veracruz (Borkent and Spinelli 2000).

**General distribution.** Neotropical. Mexico, Central America, Trinidad and Tobago, Ecuador, Brazil (Borkent and Spinelli 2007).

**Immature stages.** Unknown.

**Associated pathogens.** Unknown.

**Culicoides (Hoffmania) foxi Ortiz, 1950**

*Culicoidesfoxi* Ortiz, 1950c: 461. Type locality: Puerto Rico, Campo Tortuguero. Additional references: Forattini (1957), Wirth (1974), Wirth and Blanton (1974), Aitken et al. (1975), Huerta (1996), Borkent and Wirth (1997), Borkent and Spinelli (2000, 2007), Huerta et al. (2012, 2020).

**Distribution in Mexico.** Veracruz (Wirth and Blanton 1974), Guerrero, Oaxaca (Spinelli et al. 1993), Chiapas (Borkent and Spinelli 2007), Tabasco (Huerta et al. 2022).

**General distribution.** Neotropical. Mexico, Puerto Rico, Central and South America (Borkent and Spinelli 2007).

**Immature stages.** Unknown.

**Associated pathogens.***Leishmaniabraziliensis* (Rebêlo et al. 2016).

**Culicoides (Hoffmania) hylas Macfie, 1940**

*Culicoideshylas* Macfie, 1940: 26. Type locality: Guyana, New River. Additional references: Wirth (1974), Wirth et al. (1988), Borkent and Wirth (1997), Borkent and Spinelli (2000, 2007), Huerta et al. (2012).

**Distribution in Mexico.** Veracruz (Wirth and Blanton 1968), Oaxaca (Huerta et al. 2020).

**General distribution.** Neotropical. Mexico, Central America, Venezuela, Colombia, Ecuador, Peru, Brazil (Borkent and Spinelli 2007).

**Immature stages.** Pupa (Forattini 1957).

**Associated pathogens.** Unknown.

**Culicoides (Hoffmania) insignis Lutz, 1913**

*Culicoidesinsignis* Lutz, 1913: 51. Type locality: Brazil, Rio de Janeiro. Additional references: Wirth and Blanton (1956c, 1959, 1974), Wirth (1965, 1974), Blanton and Wirth (1979), Wirth et al. (1985, 1988), Borkent and Spinelli (2000, 2007), Borkent and Grogan (2009), Huerta et al. (2022).

*Culicoidesinamollae* Fox & Hoffman, 1944: 110, syn. Type locality: Puerto Rico.

*Culicoidespainteri* Fox, 1946: 257, syn. Type locality: Honduras.

**Distribution in Mexico.** Chiapas (Macfie 1948), Tamaulipas (Wirth and Blanton 1956c), Yucatán (Blanton and Wirth 1979), Tabasco, Veracruz (Huerta et al. 2012), Oaxaca (Huerta et al. 2020).

**General distribution.** Nearctic. USA. Neotropical. Mexico, Central and South America (Borkent and Spinelli 2007).

**Immature stages.** Larva, pupa (Forattini 1957).

**Associated pathogens.** Bluetongue virus (Tanya et al. 1992).

**Culicoides (Hoffmania) ocumarensis Ortiz, 1950**

*Culicoidesocumarensis* Ortiz, 1950b: 455. Type locality: Venezuela, Miranda, Ocumare del Tuy. Additional references: Wirth (1974), Wirth et al. (1988), Borkent and Spinelli (2000, 2007), Huerta et al. (2020).

**Distribution in Mexico.** Oaxaca, Tabasco (Spinelli et al. 1993).

**General distribution.** Neotropical. Mexico, Belize, Guatemala, El Salvador, Honduras, Nicaragua, Costa Rica, Panama, Colombia, Venezuela, Brazil (Borkent and Spinelli 2007).

**Immature stages.** Unknown.

**Associated pathogens.** Unknown.

**Culicoides (Hoffmania) palpalis Macfie, 1948**

*Culicoidespalpalis* Macfie, 1948: 78. Type locality: Mexico, Chiapas, San Cristobal. Additional references: Wirth and Blanton (1968), Wirth (1974), Wirth et al. (1988), Borkent and Wirth (1997), Borkent and Spinelli (2000, 2007).

**Distribution in Mexico.** Chiapas (Macfie 1948).

**General distribution.** Neotropical. Mexico, Belize, Guatemala, El Salvador Honduras, Nicaragua, Costa Rica, Panama, Colombia, Peru, Brazil (Borkent and Spinelli 2007).

**Immature stages.** Unknown.

**Associated pathogens.** Unknown.

**Culicoides (Hoffmania) pseudodiabolicus Fox, 1946**

*Culicoidespseudodiabolicus* Fox, 1946: 256. Type locality: Trinidad and Tobago, Cumuto Village. Additional references: Wirth (1974), Wirth et al. (1988), Borkent and Wirth (1997), Borkent and Spinelli (2000, 2007).

**Distribution in Mexico.** Veracruz (Wirth 1974), Oaxaca (Huerta et al. 2020).

**General distribution.** Neotropical. Mexico, Belize, Guatemala, El Salvador Honduras, Nicaragua, Costa Rica, Panama, Colombia, Peru, Brazil (Borkent and Spinelli 2007).

**Immature stages.** Unknown.

**Associated pathogens.** Unknown.

**Culicoides (Hoffmania) verecundus Macfie, 1948**

*Culicoidesverecundus* Macfie, 1948: 76. Type locality: Mexico, Chiapas, El Vergel. Additional references: Fox (1955), Forattini (1957), Wirth and Blanton (1959, 1968), Wirth (1974), Wirth et al. (1988), Borkent and Wirth (1997), Borkent and Spinelli (2000, 2007).

**Distribution in Mexico.** Chiapas (Macfie 1948).

**General distribution.** Neotropical. Mexico, Belize, Guatemala, El Salvador, Honduras, Nicaragua, Costa Rica, Panama, Colombia, Ecuador (Borkent and Spinelli 2007).

**Immature stages.** Unknown.

**Associated pathogens.** Unknown.

### ﻿Subgenus Macfiella Fox, 1955

*Macfiella* Fox, 1955: 217 (as subgenus of *Culicoides*). Type species: *Ceratopogonphlebotomus* Williston, by original designation.

**Culicoides (Macfiella) phlebotomus (Williston, 1896)**

*Culicoidesphlebotomus* (Williston, 1896): 281 (as *Ceratopogon*). Type locality: St. Vincent. Additional references: Forattini (1957), Wirth (1974), Wirth and Blanton (1974), Wirth et al. (1988), Borkent and Wirth (1997), Borkent and Spinelli (2000, 2007).

*Culicoidesamozonius* Macfie, 1935: 52, syn. Type locality: Brazil, Pará.

**Distribution in Mexico.** Oaxaca (Wirth and Blanton 1953), Guerrero (Vargas 1954), Sinaloa (Wirth et al. 1988), Campeche, Quintana Roo, Yucatán (Huerta et al. 2012).

**General distribution.** Neotropical. Mexico, Central America, Colombia, Ecuador, Venezuela, Jamaica, Brazil (Borkent and Spinelli 2007).

**Immature stages.** Larva, pupa (Painter 1927).

**Associated pathogens.***Mansonellaozzardi* (Nathan 1981).

**Culicoides (Macfiella) willistoni Wirth & Blanton, 1953**

*Culicoideswillistoni* Wirth & Blanton, 1953: 116. Type locality: Panama, Coclé, Rio Hato. Additional references: Wirth (1974), Wirth et al (1988), Borkent and Wirth (1997), Borkent and Spinelli (2000, 2007).

**Distribution in Mexico.** Sonora (Wirth et al. 1988).

**General distribution.** Neotropical. Mexico, Honduras, Panama (Borkent and Spinelli 2007).

**Immature stages.** Unknown.

**Associated pathogens.** Unknown.

### ﻿Subgenus Mataemyia Vargas, 1960

*Mataemyia* Vargas, 1960: 43 (as subgenus of *Culicoides*). Type species: *Culicoidesmojingaensis* Wirth and Blanton, by original designation.

**Culicoides (Mataemyia) dicrourus Wirth & Blanton, 1955**

*Culicoidesdicrourus* Wirth & Blanton, 1955b: 123. Type locality: Panama, Canal Zone, Loma Borracho. Additional references: Wirth and Blanton (1959), Wirth (1974), Wirth et al. (1988), Borkent and Spinelli (2000, 2007).

**Distribution in Mexico**. Tabasco (Huerta et al. 2022).

**General distribution.** Neotropical. Mexico, Costa Rica, Panama, Colombia, Ecuador (Borkent and Spinelli 2007).

**Immature stages.** Larva, pupa (Wirth and Soria 1981).

**Associated pathogens.** Unknown.

### ﻿Subgenus Monoculicoides Khalaf, 1954

*Monoculicoides* Khalaf, 1954: 39 (as subgenus of *Culicoides*). Type species: *Ceratopogonnubeculosus* Meigen, by original designation.

*Stigmoculicoides* Isaev, 1988: 15 (as subgenus of *Culicoides*). Type species: *Culicoidesstigma* (Meigen), by original designation.

**Culicoides (Monoculicoides) occidentalis Wirth & Jones, 1957**

*Culicoidesoccidentalis* Wirth & Jones, 1957: 21 (as subspecies of *variipennis*). Type locality: United States, California, Lake County. Additional references: Wirth and Jones (1957), Wirth (1965, 1974), Wirth et al. (1985), Borkent and Wirth (1997), Borkent and Spinelli (2000), Borkent and Grogan (2009).

**Distribution in Mexico.** Baja California, Puebla (Borkent and Spinelli 2000), Baja California Sur (Huerta et al. 2012).

**General distribution.** Nearctic. USA, Mexico (Borkent and Grogan 2009).

**Immature stages.** Larva (as *variipennis*) (Murphree and Mullen 1991), pupa (Shults and Borkent 2018).

**Associated pathogens.** Unknown.

**Culicoides (Monoculicoides) sonorensis Wirth & Jones, 1957**

*Culicoidessonorensis* Wirth & Jones, 1957: 18 (as subspecies of *variipennis*). Type locality: United States, Arizona, Cochise County. Additional references: Wirth (1965, 1974), Wirth et al. (1985, 1988), Borkent and Grogan (2009).

*Culicoidesvariipennisalbertensis* Wirth & Jones, 1957: 17, syn. Type locality: Canada.

*Culicoidesvariipennisaustralis* Wirth & Jones, 1957: 15, syn. Type locality: United States, Louisiana.

**Distribution in Mexico.** Estado de Mexico, Guerrero, Mexico City, Nuevo León, Nuevo León, Puebla, Sonora (Wirth and Jones 1957), Coahuila, Durango, Nayarit, San Luis Potosí (Huerta et al. 2012).

**General distribution.** Nearctic. USA, Mexico. Neotropical. Mexico (Borkent and Grogan 2009).

**Immature stages.** Egg (as *variipennis*) (Jones 1957), larva (as *variipennis*) (Wirth 1952), pupa (Borkent 2012; Shults et al. 2016, redescription).

**Associated pathogens.** Bluetongue virus (Price and Hardy 1954), African horse-sickness virus (Mellor et al. 1975), Epizootic hemorrhagic disease virus (Foster et al. 1977) and Vesicular stomatitis virus (Drolet et al. 2005).

**Culicoides (Monoculicoides) variipennis (Coquillett, 1901)**

*Culicoidesvariipennis* (Coquillett, 1901): 602 (as *Ceratopogon*). Type locality: United States, Virginia, Richmond. Additional references: Vargas (1945), Wirth (1965, 1974), Blanton and Wirth (1979), Wirth et al. (1985, 1988), Borkent and Spinelli (2000), Borkent and Grogan (2009), Huerta et al. (2012).

**Distribution in Mexico.** Mexico City (Hoffman 1925), Estado de México (Holbrook et al. 2000).

**General distribution.** Nearctic. USA, Mexico. Neotropical. Mexico (Borkent and Grogan 2009).

**Immature stages.** Larva (Jones 1955), pupa (Malloch 1915; Shults and Borkent 2018, redescribed).

**Associated pathogens.** Bluetongue virus (Price and Hardy 1954).

### ﻿Subgenus Oecacta Poey, 1853

*Oecacta* Poey, 1853: 238. Type species: *Oecactafurens* Poey, by monotypy.

**Culicoides (Oecacta) barbosai Wirth & Blanton, 1956**

*Culicoidesbarbosai* Wirth &Blanton, 1956a: 161. Type locality: Panama, Canal Zone, Mojinga Swamp. Additional references: Wirth (1965, 1974), Blanton and Wirth (1979), Wirth et al. (1985, 1988), Borkent and Wirth (1997), Borkent and Spinelli (2000, 2007), Borkent and Grogan (2009).

**Distribution in Mexico.** Quintana Roo (Blanton and Wirth 1979).

**General distribution.** Nearctic. USA. Neotropical. Mexico, Belize, Guatemala, El Salvador, Honduras, Nicaragua, Costa Rica, Panama, Colombia, Ecuador (Borkent and Grogan 2009).

**Immature stages.** Egg (Linley and Davies 1971), larva (Murphree and Mullen 1991), pupa (Blanton and Wirth 1979).

**Associated pathogens.***Mansonellaozzardi* (Lowrie and Raccurt 1984), *Leishmaniamexicana* (Ríos-Tostado et al. 2021).

**Culicoides (Oecacta) cancer Hogue & Wirth, l968**

*Culicoidescancer* Hogue & Wirth, 1968: 2. Type locality: Costa Rica, Puntarenas, Golfo de Nicoya, Boca de Barranca. Additional references: Wirth (1974), Wirth et al. (1988), Borkent and Wirth (1997), Borkent and Spinelli (2000, 2007).

**Distribution in Mexico.** Sinaloa (Wirth et al. 1988).

**General distribution.** Neotropical. Mexico, El Salvador, Costa Rica (Borkent and Spinelli 2007).

**Immature stages.** Larva, pupa (Hogue and Wirth 1968).

**Associated pathogens.** Unknown.

**Culicoides (Oeacta) furens Poey, 1853**

*Culicoidesfurens* (Poey, 1853): 236. Type locality: Cuba. Additional references: Hoffman (1925), Fox (1955), Forattini (1957), Wirth and Blanton (1959, 1974), Wirth (1965, 1974), Blanton and Wirth (1979), Wirth et al. (1985, 1988), Borkent and Wirth (1997), Borkent and Spinelli (2000, 2007), Borkent and Grogan (2009).

*Ceratopogonmaculithorax* (Williston, 1896): 277, syn. Type locality: St. Vincent.

*Culicoidesdovei* Hall, 1932: 88, syn. Type locality: United States, Georgia.

*Culicoidesbirabeni* Cavalieri, 1966: 59, syn. Type locality: Venezuela.

**Distribution in Mexico.** Veracruz (Townsend 1897), Tabasco (Hoffman 1925), Campeche (Barbosa 1947), Sinaloa (Vargas 1945), Guerrero, Sonora, Tamaulipas, Yucatán (Wirth and Blanton 1974), Baja California, Hidalgo, Nayarit (Huerta et al. 2012).

**General distribution.** Nearctic. USA, Mexico. Neotropical. Mexico, Central America, Caribbean islands, Colombia, Venezuela, Ecuador, Brazil (Borkent and Grogan 2009).

**Immature stages.** Larva, pupa (Painter 1927).

**Associated pathogens.***Mansonellaozzardi* (Buckley 1934), *Tetrapetalonemamarmosetae* (Lowrie et al. 1978), *Leishmaniamexicana* (Ríos-Tostado et al. 2021).

### ﻿Subgenus Selfia Khalaf, 1954

*Selfia* Khalaf, 1954: 38 (as subgenus of *Culicoides*). Type species: *Culicoideshieroglyphicus* Malloch, by original designation.

**Culicoides (Selfia) hieroglyphicus Malloch, 1915**

*Culicoideshieroglyphicus* Malloch, 1915: 297. Type locality: United States, Arizona, Graham Mountains, Ash Creek. Additional references: Atchley (1967), Wirth (1965, 1974), Wirth et al. (1985, 1988), Borkent and Wirth (1997), Borkent and Spinelli (2000), Borkent and Grogan (2009).

**Distribution in Mexico.** Baja California, Sonora (Atchley 1970), Durango (Wirth et al. 1988).

**General distribution.** Nearctic. USA, Mexico (Borkent and Grogan 2009).

**Immature stages.** Larva (Atchley 1970), pupa (Jones 1961).

**Associated pathogens.** Unknown.

**Culicoides (Selfia) multipunctatus Malloch, 1915**

*Culicoidesmultipunctatus* Malloch, 1915: 296. Type locality: United States, Illinois, Urbana. Additional references: Wirth (1965), Wirth et al. (1985), Borkent and Spinelli (2000), Borkent and Grogan (2009).

**Distribution in Mexico.** Tamaulipas (Atchley 1970), Morelos (Wirth et al. 1988).

**General distribution.** Nearctic. USA, Mexico (Borkent and Grogan 2009).

**Immature stages.** Pupa (Jones 1961).

**Associated pathogens.** Unknown.

### ﻿Subgenus unplaced, *acotylus* species group

***Culicoidesacotylus* Lutz, 1913**

*Culicoidesacotylus* Lutz, 1913: 69. Type locality: Brazil, Mato Grosso, Rio Tapajós. Additional references: Fox (1955), Forattini (1957), Wirth and Blanton (1959), Wirth (1974), Aitken et al. (1975), Wirth et al. (1988), Borkent and Wirth (1997), Borkent and Spinelli (2000, 2007).

*Culicoidespanamericanus* Fox, 1947: 90, syn. Type locality: Mexico, Mexico City (formerly Distrito Federal).

**Distribution in Mexico.** Mexico City (Fox, 1947, as *panamericanus*).

**General distribution.** Neotropical. Mexico, Honduras, Panama, Venezuela, Trinidad and Tobago, Surinam, Brazil (Borkent and Spinelli 2007).

**Immature stages.** Unknown.

**Associated pathogens.** Unknown.

### ﻿Subgenus unplaced, *daedalus* species group

***Culicoidescrescentis* Wirth & Blanton, 1959**

*Culicoidescrescentis* Wirth & Blanton, 1959: 317. Type locality: Panama, Canal Zone, Mojinga Swamp. Additional references: Wirth (1974), Wirth et al. (1988), Borkent and Wirth (1997), Borkent and Spinelli (2000, 2007).

**Distribution in Mexico.** Chiapas (Wirth and Blanton 1959).

**General distribution.** Neotropical. Mexico, Belize, Guatemala, El Salvador, Honduras, Nicaragua, Costa Rica, Panama, Colombia, Argentina (Borkent and Spinelli 2007).

**Immature stages.** Unknown.

**Associated pathogens.** Unknown.

***Culicoidesdaedalus* Macfie, 1948**

*Culicoidesdaedalus* Macfie, 1948: 83. Type locality: Mexico, Chiapas, El Vergel. Additional references: Fox (1955), Forattini (1957), Wirth and Blanton (1959), Wirth (1965, 1974), Wirth et al. (1985, 1988), Borkent and Wirth (1997), Borkent and Spinelli (2000, 2007), Borkent and Grogan (2009).

**Distribution in Mexico.** Chiapas (Macfie 1948), Veracruz (Huerta et al. 2012).

**General distribution**. Nearctic. USA, Mexico. Neotropical. Mexico, Belize, Guatemala, El Salvador, Honduras, Nicaragua, Costa Rica, Panama, Colombia (Borkent and Spinelli 2007).

**Immature stages.** Unknown.

**Associated pathogens.** Unknown.

***Culicoidespampoikilus* Macfie, 1948**

*Culicoidespampoikilus* Macfie, 1948: 79. Type locality: Mexico, Chiapas, El Vergel. Additional references: Fox (1955), Forattini (1957), Wirth and Blanton (1959), Atchley (1967), Wirth (1974), Wirth et al. (1985, 1988), Borkent and Wirth (1997), Borkent and Spinelli (2000, 2007), Borkent and Grogan (2009).

Culicoidesdominicii Ortiz, 1951: 7, syn. Type locality: Venezuela.

**Distribution in Mexico.** Chiapas (Macfie 1948), Oaxaca (Vargas 1945; Vargas 1954), Veracruz (Huerta et al. 2012).

**General distribution.** Nearctic. USA. Neotropical. Mexico, Belize, Guatemala, El Salvador, Honduras, Nicaragua, Costa Rica, Panama, Colombia, Venezuela (Borkent and Spinelli 2007).

**Immature stages.** Unknown.

**Associated pathogens.** Unknown.

### ﻿Subgenus unplaced, *eublepharus* species group

***Culicoideseublepharus* Macfie, 1948**

*Culicoideseublepharus* Macfie, 1948: 86. Type locality: Guyana. Additional references: Wirth (1974), Wirth et al. (1988), Borkent and Spinelli (2000, 2007).

*Culicoidestransferrans* Ortiz, 1953: 801, syn. Type locality: Venezuela.

**Distribution in Mexico.** Chiapas (Wirth et al. 1988).

**General distribution.** Neotropical. Mexico, Costa Rica, Panama, Colombia, Ecuador, Venezuela, Brazil (Borkent and Spinelli 2007).

**Immature stages.** Unknown.

**Associated pathogens.** Unknown.

***Culicoidespropriipennis* Macfie, 1948**

*Culicoidespropriipennis* Macfie, 1948: 84. Mexico, Chiapas, San Cristóbal de las Casas: Additional references: Fox (1955), Forattini (1957), Wirth and Blanton (1959), Wirth (1974), Wirth et al. (1988), Borkent and Wirth (1997), Borkent and Spinelli (2000, 2007).

**Distribution in Mexico.** Chiapas (Macfie 1948).

**General distribution.** Neotropical. Mexico, Belize, Guatemala, El Salvador, Honduras, Nicaragua, Costa Rica, Panama, Colombia, Ecuador, Venezuela, Brazil (Borkent and Spinelli 2007).

**Immature stages.** Unknown.

**Associated pathogens.** Unknown.

***Culicoidesrangeli* Ortiz & Mirsa, 1952**

*Culicoidesrangeli* Ortiz & Mirsa, 1952: 126. Type locality. Venezuela, Miranda, Los Chorros. Additional references: Forattini (1957), Wirth and Blanton (1959), Wirth (1974, as *donajii*), Aitken et al. (1975), Wirth et al. (1988), Borkent and Wirth (1997), Borkent and Spinelli (2000, 2007).

*Culicoidesdonajii* Vargas, 1954: 28, syn. Type locality: Mexico, Oaxaca.

*Culicoidespatupalpis* Wirth & Blanton, 1959: 421, syn. Type locality: Panama.

**Distribution in Mexico.** Oaxaca (Vargas 1954, as *donajii*).

**General distribution.** Neotropical. Mexico, Central America, Colombia, Ecuador, Bolivia, Venezuela, Trinidad and Tobago, Brazil (Borkent and Spinelli 2007).

**Immature stages.** Unknown.

**Associated pathogens.** Unknown.

### ﻿Subgenus unplaced, *fluvialis* species group

***Culicoidescastillae* Fox, 1946**

*Culicoidescastillae* Fox, 1946: 251. Type locality: Honduras, Puerto Castilla. Additional references: Wirth (1974), Wirth et al. (1988), Borkent and Wirth (1997), Borkent and Spinelli (2000, 2007).

*Culicoidesgibsoni* Wirth, 1952: 246, syn. Type locality: Guatemala.

*Culicoidesflochabonnenci* Ortiz & Mirsa, 1952: 267, syn. Type locality: Venezuela.

**Distribution in Mexico.** Michoacán (Huerta et al. 2012).

**General distribution.** Neotropical. Mexico, Guatemala, El Salvador, Honduras, Nicaragua, Costa Rica, Panama, Colombia, Ecuador, Venezuela, Trinidad and Tobago (Borkent and Spinelli 2007).

**Immature stages.** Unknown.

**Associated pathogens.** Unknown.

***Culicoidesleopoldoi* Ortiz, 1951**

*Culicoidesleopoldoi* Ortiz, 1951: 579. Type locality: Venezuela, Ocumare del Tuy. Additional references: Vargas (1954), Forattini (1957), Wirth and Blanton (1959), Wirth (1974), Aitken et al. (1975), Wirth et al. (1988), Borkent and Wirth (1997), Borkent and Spinelli (2000, 2007).

**Distribution in Mexico.** Oaxaca (Huerta et al. 2012), Tabasco (Huerta et al. 2022).

**General distribution.** Neotropical. Mexico, Central America, Colombia, Ecuador, Venezuela, Trinidad and Tobago, Bolivia, Argentina (Borkent and Spinelli 2007).

**Immature stages.** Unknown.

**Associated pathogens.** Unknown.

### ﻿Subgenus unplaced, *leoni* species group

***Culicoidesgabaldoni* Ortiz, 1954**

*Culicoidesgabaldoni* Ortiz, 1954: 221. Type locality: Venezuela, Yaracuy, San Felipe. Additional references: Wirth (1974), Wirth and Blanton (1974), Borkent and Wirth (1997), Borkent and Spinelli (2000, 2007), Huerta et al. (2022).

**Distribution in Mexico.** Tabasco (Wirth et al. 1988), Oaxaca, Veracruz (Huerta et al. 2012).

**General distribution.** Neotropical. Mexico, Central and South America (Borkent and Spinelli 2007).

**Immature stages described.** Unknown.

**Pathogens associated.** Unknown.

***Culicoidesglabellus* Wirth & Blanton, 1956**

*Culicoidesglabellus* Wirth & Blanton, 1956d: 47. Type locality: Panama, Boca del Toro, Almirante. Additional references: Wirth (1974), Wirth et al. (1988), Borkent and Wirth (1997), Borkent and Spinelli (2000, 2007), Huerta et al. (2022).

**Distribution in Mexico.** Oaxaca (Huerta et al. 2020).

**General distribution.** Neotropical. Mexico, Honduras, Nicaragua, Costa Rica, Panama, Colombia, Ecuador, Trinidad and Tobago, Venezuela, Brazil (Borkent and Spinelli 2007).

**Immature stages.** Unknown.

**Associated pathogens.** Unknown.

***Culicoidesleoni* Barbosa, 1952**

*Culicoidesleoni* Barbosa, 1952: 17. Type locality: Ecuador, Santo Domingo. Additional references: Forattini (1957), Wirth (1974), Wirth et al. (1988), Borkent and Wirth (1997), Borkent and Spinelli (2000, 2007).

**Distribution in Mexico.** Veracruz (Huerta et al. 2012).

**General distribution.** Neotropical. Mexico, Ecuador (Borkent and Spinelli 2007).

**Immature stages.** Unknown.

**Associated pathogens.** Unknown.

### ﻿Subgenus unplaced, *limai* species group

***Culicoidesluglani* Jones & Wirth, 1958**

*Culicoidesluglani* Jones & Wirth, 1958: 89. Type locality: United States, Texas, Kerr County. Additional references: Wirth (1965, 1974), Atchley (1967), Wirth et al. (1985, 1988), Borkent and Wirth (1997), Borkent and Spinelli (2000), Borkent and Grogan (2009).

**Distribution in Mexico.** Baja California, Sonora, Tamaulipas (Wirth 1974).

**General distribution.** Nearctic. USA, Mexico (Borkent and Grogan 2009).

**Immature stages.** Unknown.

**Associated pathogens.** Unknown.

### ﻿Subgenus unplaced, *mohave* species group

***Culicoidesbajensis* Wirth & Moraes, 1979**

*Culicoidesbajensis* Wirth & Moraes, 1979: 291. Type locality: Mexico, Baja California Sur, Penjamo. Additional references: Wirth et al. (1985, 1988), Borkent and Wirth (1997), Borkent and Spinelli (2000), Borkent and Grogan (2009).

**Distribution in Mexico.** Baja California Sur, Sonora (Wirth and Moraes 1979).

**General distribution.** Nearctic. USA, Mexico (Borkent and Grogan 2009).

**Immature stages.** Unknown.

**Associated pathogens.** Unknown.

***Culicoideshoguei* Wirth & Moraes, 1979**

*Culicoideshoguei* Wirth & Moraes, 1979: 293. Type locality: United States, California, Orange County, Seal Beach Weapons Station. Additional references: Wirth et al. (1985, 1988), Borkent and Wirth (1997), Borkent and Spinelli (2000), Borkent and Grogan (2009).

**Distribution in Mexico.** Baja California (Wirth and Moraes 1979).

**General distribution.** Nearctic. USA, Mexico (Borkent and Grogan 2009).

**Immature stages.** Unknown.

**Associated pathogens.** Unknown.

***Culicoidesmohave* Wirth, 1952**

*Culicoidesmohave* Wirth, 1952: 187. Type locality: United States, California, San Bernardino County, Vidal: Additional references: Wirth (1965, 1974), Wirth and Moraes (1979), Wirth et al. (1985,^1988^), Borkent and Wirth (1997), Borkent and Spinelli (2000), Borkent and Grogan (2009).

**Distribution in Mexico.** Baja California (Wirth 1952).

**General distribution.** Nearctic. USA, Mexico (Borkent and Grogan 2009).

**Immature stages.** Unknown.

**Associated pathogens.** Unknown.

***Culicoideswoodruffi* Spinelli & Huerta, 2015**

*Culicoideswoodruffi* Spinelli & Huerta, 2015: 821. Type locality: Mexico, Morelos.

**Distribution in Mexico.** Morelos (Spinelli and Huerta 2015).

**General distribution.** Endemic. Mexico (Spinelli and Huerta 2015).

**Immature stages.** Unknown.

**Associated pathogens.** Unknown.

### ﻿Subgenus unplaced, *reticulatus* species group

***Culicoideslanei* Ortiz, 1950**

*Culicoideslanei* Ortiz, 1950a: 431. Type locality: Panama, Cerro Zefa. Additional references: Wirth (1974), Aitken et al. (1975), Wirth et al. (1988), Borkent and Wirth (1997), Borkent and Spinelli (2000, 2007).

**Distribution in Mexico.** Veracruz (Wirth et al. 1988).

**General distribution.** Neotropical. Mexico, Honduras, Costa Rica, Panama, Venezuela, Trinidad, Brazil (Borkent and Spinelli 2007).

**Immature stages.** Unknown.

**Associated pathogens.** Unknown.

### ﻿Subgenus unplaced, *stigmalis* species group

***Culicoidesstigmalis* Wirth, 1952**

*Culicoidesstigmalis* Wirth, 1952: 245. Type locality: Guatemala, Chimaltenango, San Pedro Yepocapa. Additional references: Wirth (1974), Wirth et al. (1988), Borkent and Spinelli (2000, 2007).

**Distribution in Mexico.** Oaxaca (Vargas 1953b), Veracruz (Huerta et al. 2012).

**General distribution.** Neotropical. Mexico, Guatemala, Costa Rica, Panama (Borkent and Spinelli 2007).

**Immature stages.** Unknown.

**Associated pathogens.** Unknown.

### ﻿Subgenus unplaced, *stonei* species group

***Culicoidesmelleus* (Coquillett, 1901)**

*Culicoidesmelleus* (Coquillett, 1901): 604 (as *Ceratopogon*). Type locality: United States, Florida, Lake Worth. Additional references: Wirth (1965, 1974), Wirth et al. (1985), Borkent and Spinelli (2000, 2007), Borkent and Grogan (2009).

**Distribution in Mexico.** Baja California (Wirth et al. 1988).

**General distribution.** Nearctic. Canada, USA, Mexico (Borkent and Grogan 2009).

**Immature stages.** Unknown.

**Associated pathogens.** Unknown.

***Culicoideswerneri* Wirth & Blanton, 1971**

*Culicoideswerneri* Wirth & Blanton, 1971b: 463. Type locality: United States, Arizona, Quitobaquito, Pima County. Additional references: Wirth et al. (1985), Borkent and Spinelli (2000), Borkent and Grogan (2009).

**Distribution in Mexico.** Sonora (Wirth and Blanton 1971b).

**General distribution.** Nearctic. USA, Mexico (Borkent and Grogan 2009).

**Immature stages.** Unknown.

**Associated pathogens.** Unknown.

### ﻿Species of Culicoides unplaced to subgenus or species >group

***Culicoidesalbomaculus* Root & Hoffman, 1937**

*Culicoidesalbomaculus* Root & Hoffman, 1937 (as *albumacula*): 164. Type locality: Mexico, Mexico City, San Jacinto. Additional references: Vargas (1945, 1949), Wirth (1974), Wirth et al. (1988), Borkent and Wirth (1997), Borkent and Spinelli (2000).

**Distribution in Mexico.** Mexico City (Root and Hoffman 1937).

**General distribution.** Endemic. Mexico (Borkent and Spinelli 2000).

**Immature stages.** Unknown.

**Associated pathogens.** Unknown.

***Culicoidesarubae* Fox & Hoffman, 1944**

*Culicoidesarubae* Fox & Hoffman, 1944: 109. Type locality: Aruba. Additional references: Forattini (1957), Wirth (1965, 1974), Wirth and Blanton (1974), Wirth et al. (1985, 1988), Borkent and Wirth (1997), Borkent and Spinelli (2000, 2007), Borkent and Grogan (2009).

**Distribution in Mexico.** Tamaulipas (Vargas 1954), Campeche, Guerrero (Wirth et al. 1988), Veracruz, Yucatán (Huerta et al. 2012).

**General distribution.** Nearctic. USA, Mexico. Neotropical. Mexico, Aruba, Grenada, Colombia, Venezuela (Borkent and Spinelli 2007).

**Immature stages.** Unknown.

**Associated pathogens.** Unknown.

***Culicoidesneghmei* Vargas, 1955**

*Culicoidesneghmei* Vargas, 1955: 673. Type locality: Mexico, Puebla, Atlixco. Additional references: Wirth (1974), Borkent and Wirth (1997), Borkent and Spinelli (2000).

**Distribution in Mexico.** Puebla (Vargas 1955).

**General distribution.** Endemic. Mexico (Borkent and Spinelli 2000).

**Immature stages.** Unknown.

**Associated pathogens.** Unknown.

***Culicoidespropinquus* Macfie, 1948**

*Culicoidespropinquus* Macfie, 1948: 81. Type locality: Mexico, Chiapas, San Cristobal. Additional references: Vargas (1949), Fox (1955), Forattini (1957), Wirth (1974), Wirth et al. (1988), Borkent and Wirth (1997), Borkent and Spinelli (2000).

**Distribution in Mexico.** Chiapas (Macfie 1948).

**General distribution.** Endemic. Mexico (Borkent and Spinelli 2000).

**Immature stages.** Unknown.

**Associated pathogens.** Unknown.

### ﻿Keys to the subgenera and species groups of *Culicoides* of Mexico

**Table:** 

1	One spermatheca	**2**
–	Two or three spermathecae	**6**
2	Spermatheca irregular or U-shaped; pigmentation of wing mostly white with isolated black spots; male parameres fused	***Monoculicoides***
–	Spermatheca oval or pyriform; pigmentation of wing variable; male parameres separate	**3**
3	Wing with abundant macrotrichia; flagellomeres 10–12 with coeloconica sensilla	**4**
–	Wing with sparse microtrichia limited to the distal part; flagellomeres 10–12 without coeloconica sensilla	**5**
4	Wing cells m_1_, m_2_ and distal part of r_3_ with small white spots; the two post-stigmatic spots separate; flagellomere 13 without coeloconica sensilla	***eublepharus* species group**
–	Wing cells m_1_, m_2_ and distal part of r_3_ with large white spots, nearly filling the cells; the two post-stigmatic spots fused; flagellomere 13 with coeloconica sensilla	***Beltranmyia***
5	Small species, wing length < .8 mm; cell m_2_ and distal part of anal cell with two white spots	***leoni* species group**
–	Large species, wing length > .8 mm; cell m_2_ and distal part of anal cell with one white spot	***fluvialis* species group**
6	Legs with tarsomere 4 cordiform, wider than long	***Macfiella***
–	Legs with tarsomere 4 cylindrical	**7**
7	Wing unspotted	**8**
–	Wings with black or light spots, evident or diffuse, variable pattern	**9**
8	Three unsclerotized or slightly sclerotized spermathecae	***Selfia***
–	Two sclerotized spermathecae	***stonei* species group**
9	Wing pigmentation diffuse, cell r_2_ partially or completely included on black spot	**10**
–	Wing pigmentation conspicuous, with multiple white or black spots, variable pattern	**11**
10	Small species, wing length < .85 mm; wing with cell r_2_ small, with a conspicuous black spot encompassing the apical portion of r_1_ and basal portion of r_2_; distal portion of r_2_ pale	***Avaritia***
–	Large species, wing length > .85 mm; wing with cell r_2_ very large, about three times as long as broad, completely dark	***stigmalis* species group**
11	Wing with second radial cell completely or partially included in a white spot	**12**
–	Wing with second radial cell completely included in a black spot	**14**
12	Wings with predominant, extensive, interconnected black spots; cell m_4_ with a white spot at the base of the Cu-M_4_ bifurcation and another white spot in front of it of variable size	***Hoffmania***
–	Wings with predominant, extensive, interconnected white spots; cell m_4_ with a dark spot at base of the Cu-M_4_ bifurcation	**13**
13	Tibial comb with 6 spines	***Culicoides* s. str.**
–	Tibial comb with 4 spines	***Anilomyia***
14	Vein r-m dark	**15**
–	Vein r-m pale	**16**
15	Flagellomeres 9–13 with coeloconica sensilla	***Glaphiromyia***
–	Flagellomeres 9–13 without coeloconica sensilla	***acotylus* species group**
16	Vein M_2_ without a white spot straddling the middle portion	**17**
–	Vein M_2_ with a white spot straddling the middle portion	**19**
17	Vein M_1_ and M_2_ dark; cell m_2_ with one spot	***Haematomyidium* and *mohave* species group**
–	Vein M_1_ and M_2_ on white bands; cell m_2_ with more than one spot	**18**
18	Cell m_1_ with one white spot distal to the medial bifurcation	***Mataemyia***
–	Cell m_1_ with two white spots distal to the medial bifurcation	***reticulatus* species group**
19	Scutum without a pattern of numerous black punctiform dots, variable pattern	**20**
–	Scutum with a pattern of numerous black punctiform dots	***Oecacta***
20	Anal cell with three white spots arranged in a triangular pattern	***Amossovia***
–	Anal cell with one or two white spots	**21**
21	Flagellomeres 9–13 with coeloconica sensilla	**22**
–	Flagellomeres 9–13 without coeloconica sensilla	***Diphaomyia***
22	Vein M_1_ without a white spot straddling the middle portion; cell r_3_ with distal white spot large, nearly filling de cell	***limai* species group**
–	Vein M_1_ with a white spot straddling the middle portion; cell r_3_ with distal white spot small	***Drymodesmyia* and *daedalus* species group**

### ﻿Species list by state

**Aguascalientes**: without records.

**Baja California**: *Culicoidesarizonensis*, *C.boydi*, *C.copiosus*, *C.furens*, *C.haematopotus*, *C.hieroglyphicus*, *C.hoguei*, *C.insolatus*, *C.kettlei*, *C.luglani*, *C.melleus*, *C.mohave*, *C.occidentalis*, *C.panamensis*, *C.ryckmani*, *C.sitiens*, *C.torridus*.

**Baja California Sur**: *Culicoidesbajensis*, *C.cacticola*, *C.cochisensis*, *C.occidentalis*, *C.oklahomensis*.

**Campeche**: *Culicoidesarubae*, *C.furens*, *C.phlebotomus*.

**Chiapas**: *Culicoidescrescentis*, *C.daedalus*, *C.diabolicus*, *C.elutus*, *C.eublepharus*, *C.filariferus*, *C.foxi*, *C.haematopotus*, *C.insignis*, *C.iriartei*, *C.jamaicensis*, *C.luteovenus*, *C.neopulicaris*, *C.palpalis*, *C.pampoikilus*, *C.panamensis*, *C.paraensis*, *C.poikilonotus*, *C.propinquuos*, *C.propriipennis*, *C.pusilloides*, *C.pusillus*, *C.verecundus*.

**Chihuahua**: without records.

**Coahuila**: *Culicoidescrepuscularis*, *C.sonorensis*.

**Colima**: without records.

**Mexico City**: *Culicoidesacotylus*, *C.albomaculus*, *C.bakeri*, *C.copiosus*, *C.crepuscularis*, *C.dampfi*, *C.haematopotus*, *C.luteovenus*, *C.scopus*, *C.sonorensis*, *C.variipennis*.

**Durango**: *Culicoideshieroglyphicus*, *C.sonorensis*.

**Guanajuato**: without records.

**Guerrero**: *Culicoidesarubae*, *C.blantoni*, *C.foxi*, *C.furens*, *C.haematopotus*, *C.jamaicensis*, *C.neopulicaris*, *C.phlebotomus*, *C.scopus*, *C.sonorensis*, *C.variipennis*, *C.wirthomyia*.

**Hidalgo**: *Culicoidesfurens*, *C.neopulicaris*, *C.nigrigenus*.

**Jalisco**: *Culicoidesjamaicensis*.

**Estado de México**: *Culicoidesjamaicensis*, *C.neopulicaris*, *C.panamensis*, *C.sonorensis*, *C.variipennis*.

**Michoacán**: *Culicoidescastillae*, *C.parascopus*, *C.rulfoi*.

**Morelos**: *Culicoidesblantoni*, *C.crepuscularis*, *C.multipunctatus*, *C.neopulicaris*, *C.panamensis*, *C.pseudodecor*, *C.woodruffi*.

**Nayarit**: *Culicoideseadsi*, *C.furens*, *C.panamensis*, *C.sonorensis*.

**Nuevo León**: *Culicoidesbutleri*, *C.ousairani*, *C.sonorensis*, *C.variipennis*.

**Oaxaca**: *Culicoidesbaueri*, *C.debilipalpis*, *C.diabolicus*, *C.elutus*, *C.foxi*, *C.gabaldoni*, *C.ginesi*, *C.glabellus*, *C.hylas*, *C.insignis*, *C.jamaicensis*, *C.leopoldoi*, *C.luteovenus*, *C.neopulicaris*, *C.ocumarensis*, *C.pampoikilus*, *C.phlebotomus*, *C.pseudodiabolicus*, *C.pusillus*, *C.rangeli*, *C.stigmalis*, *C.variipennis*.

**Puebla**: *Culicoidesbaueri*, *C.blantoni*, *C.haematopotus*, *C.neghmei*, *C.occidentalis*, *C.sonorensis*.

**Querétaro**: without records.

**Quintana Roo**: *Culicoidesbarbosai*, *C.paraensis*, *C.phlebotomus*.

**San Luis Potosí**: *Culicoidesblantoni*, *C.eadsi*, *C.neopulicaris*, *C.paraensis*, *C.sonorensis*.

**Sinaloa**: *Culicoidesblantoni*, *C.cancer*, *C.furens*, *C.phlebotomus*.

**Sonora**: *Culicoidesbajensis*, *C.cacticola*, *C.crepuscularis*, *C.eadsi*, *C.furens*, *C.hieroglyphicus*, *C.luglani*, *C.oklahomensis*, *C.sonorensis*, *C.werneri*, *C.willistoni*.

**Tabasco**: *Culicoidesblantoni*, *C.foxi*, *C.furens*, *C.gabaldoni*, *C.insignis*, *C.leopoldoi*, *C.ocumarensis*, *C.paraensis*, *C.poikilonotus*, *C.pusillus*.

**Tamaulipas**: *Culicoidesarubae*, *C.blantoni*, *C.furens*, *C.hayesi*, *C.insignis*, *C.luglani*, *C.multipunctatus*.

**Tlaxcala**: without records.

**Veracruz**: *Culicoidesarubae*, *C.blantoni*, *C.crepuscularis*, *C.daedalus*, *C.debilipalpis*, *C.diabolicus*, *C.filariferus*, *C.fortinensis*, *C.foxi*, *C.furens*, *C.gabaldoni*, *C.haematopotus*, *C.hylas*, *C.iriartei*, *C.jamaicensis*, *C.lanei*, *C.leoni*, *C.luteovenus*, *C.neopulicaris*, *C.nigrigenus*, *C.pampoikilus*, *C.panamensis*, *C.paraensis*, *C.poikilonotus*, *C.pseudodecor*, *C.pseudodiabolicus*, *C.pusillus*, *C.stigmalis*.

**Yucatán**: *Culicoidesarubae*, *C.eadsi*, *C.furens*, *C.insignis*, *C.jamaicensis*, *C.loughnani*, *C.neopulicaris*, *C.phlebotomus*.

**Zacatecas**: without records.

## ﻿Conclusions

Expanding and updating the knowledge of insect vectors is essential for the creation, implementation, and improvement of surveillance and population control programs. The number of *Culicoides* species present in Mexico represents 6% of the known species worldwide, 57% of the Nearctic species and 29% of the Neotropical species. In addition, 11 species are endemic. These endemic species have adapted and diversified according to the topography, soil, and altitudinal gradients of the country and are concentrated in some states such as Mexico City, Michoacán, Veracruz, and Chiapas where more *Culicoides* endemics are known in areas 2,000 meters above sea level. However, the alteration of natural ecosystems by human activities and the scarcity of updated data makes the actual distribution of endemic *Culicoides* species uncertain. Therefore, it is essential to understand the processes that originate and sustain diversity in these areas, which are subject to rapid changes in climate and habitats. Mexico City is a special case, as it harbors more endemic *Culicoides* species, but it is also an area with strong anthropogenic and demographic pressure, which generates uncertainty about the current distribution of these species. This is especially important in sites such as Chapultepec and San Jacinto.

The Nearctic Region includes North America, covering arid and temperate zones in northern Mexico, such as Baja California, Chihuahua, and Nuevo León, as well as the central region of Mexico, which includes Mexico City and Puebla. On the other hand, the Neotropical Region extends through the tropical and subtropical zones of southern Mexico, and a large part of the coastal region of the Pacific and Gulf of Mexico.

The distribution of *Culicoides* species in Mexico is classified into four main categories: Nearctic, Neotropical, broad distribution in the New World, and endemic. Of the species present in the country, 50% are distributed in the Nearctic Region, 77% in the Neotropical Region, 27% in both regions, and 13% are endemic. A wide variety of species distribution can be observed. For example, the subgenera C. (Amossovia) and C. (Monoculicoides) are predominant in the Nearctic Region, while C. (Drimodesmyia) has a significant presence in both the Neotropical and Nearctic Regions.

On the other hand, the subgenera C. (Anilomyia), C. (Avaritia), C. (Culicoides) and C. (Diphaomyia) have a predominant distribution in the Neotropical Region. Although C. (Hoffmania) is also common in this Region, the presence of *C.insignis* has been recorded in the Nearctic and represents an important health risk. In addition, C. (Glaphiromyia) is a subgenus mainly endemic to Mexico, which makes it of special interest from a biogeographical perspective since species of this subgenus have been described in the transition zone of central Mexico.

Of Mexico’s 32 states, *Culicoides* species have been recorded in only 25. Veracruz and Chiapas had the highest richness of biting midges. The subgenus Drymodesmyia is the best represented in the country with 14 species recorded, followed by the subgenus Hoffmania, represented by nine species. It should be noted that most of the records made in the country are the result of collection events more than half a century old and few records have been made in recent years; in addition, there are species that have not been collected since they were recorded; thus, the occurrence and distribution of several species should be reevaluated.

In general, the immature stages of *Culicoides* species are largely unknown and represent an important potential area of study. The immature stages of 30% of species present in Mexico are known. The egg stage is known for 5.8% of the species, while both larval and pupal stages for 27%. On the other hand, 15 species (17.4%) have been associated with different pathogens and therefore represent a potential risk as vectors in the country. Of these, eight species were associated with viruses, among which *C.sonorensis*, *C.insignis* and *C.paraensis* stand out for their greatest impact on human and animal health. The presence of these species in the country poses a greater health risk; therefore, it is vital to increase surveillance efforts to prevent possible disease outbreaks, especially in regions of high susceptibility, such as those with high livestock production. In addition, six species were associated with the transmission of nematodes and six species with protozoa, particularly Haemosporida.

The dichotomous keys presented in this work are the first to specifically focus on the *Culicoides* fauna of Mexico. Previously, it was necessary to consult several studies to identify the species present in the country. However, since the current subgeneric classification of *Culicoides* species could include inconsistencies and is in urgent need of revision, it is likely that these keys should help update the knowledge of the genus in the country.

Lastly, it is important to note that due to the physiographic, climatic, and topographic characteristics of the country, the great variety of ecosystems with conditions like those of other neotropical countries, as well as the lack of systematic and faunistic studies that address the spatial and temporal changes of the group, it can be inferred that the species richness of *Culicoides* in the country is far from being elucidated.

## ﻿Additional information

### Author ORCIDs

Alejandro Mendez-Andrade https://orcid.org/0000-0003-4074-5704

Sergio Ibáñez-Bernal https://orcid.org/0000-0002-3182-6134

### Data availability

All of the data that support the findings of this study are available in the main text or Supplementary Information.
